# Prognostic value of association of OCT4 with LEF1 expression in esophageal squamous cell carcinoma and their impact on epithelial‐mesenchymal transition, invasion, and migration

**DOI:** 10.1002/cam4.1641

**Published:** 2018-07-04

**Authors:** Yue Zhao, Chunguang Li, Lei Huang, Shuai Niu, Qijue Lu, Dejun Gong, Shengdong Huang, Yang Yuan, Hezhong Chen

**Affiliations:** ^1^ Department of Thoracic Surgery Changhai Hospital Second Military Medical University Shanghai China; ^2^ Department of Vascular Surgery Beijing Shijitan Hospital Capital Medical University Beijing China; ^3^ Institute of Cardiothoracic Surgery Changhai Hospital Second Military Medical University Shanghai China

**Keywords:** epithelial‐mesenchymal transition, esophageal squamous cell carcinoma, LEF1, OCT4, prognosis

## Abstract

Esophageal squamous cell carcinoma (ESCC) is a malignant disease with poor prognosis. Because of early metastasis prior to diagnosis and therapeutic resistance, ESCC has become one of the leading causes of cancer‐related death. Here, we investigated the clinicopathological significance of the association of octamer‐binding transcription factor 4 (OCT4) with lymphoid enhancer‐binding factor 1 (LEF1) expression and the potential molecular mechanism in the epithelial‐mesenchymal transition (EMT), invasion, and migration of ESCC. The expression of OCT4 and LEF1 was detected via immunohistochemistry analysis. High levels of LEF1 expression were observed in 95 ESCC specimens and were obviously associated with aberrant clinicopathological features and poor patient prognosis. Our previous study showed that OCT4 expression level is elevated in ESCC, and statistical analysis showed that the elevated expression of OCT4 and LEF1 in ESCC was significantly associated with histologic grade, lymph node metastasis, TNM stage, and poor patient prognosis. The specific inhibition of OCT4 expression via a lentivirus encoding OCT4‐shRNA (LV‐shOCT4) in Eca109 cells led to decreased levels of OCT4 and LEF1 in vitro. Additionally, we applied a rescue strategy by infecting LV‐shOCT4 Eca109 cells with a LEF1 overexpression plasmid (p‐LEF1) and detected changes in EMT, migration, and invasion. Unsurprisingly, the p‐LEF1 group exhibited greater EMT, invasion, and migration than did the LV‐shOCT4 and negative control groups. This study demonstrates for the first time the relationship between OCT4 and LEF1 expression. The combination of high expression of OCT4 and LEF1 was associated with clinicopathological features of atypical patients, and this combination might be an ideal prognostic factor in ESCC. OCT4 positively regulated LEF1 expression, and LEF1 mediated the effects of OCT4 in cancer cell EMT, invasion, and migration. The data presented here suggest that the inhibition of OCT4‐LEF1 signaling may be a new therapeutic target for the treatment of ESCC.

## 1 INTRODUCTION

Esophageal squamous cell carcinoma (ESCC) is the eighth most prevalent malignancy cancer worldwide and is one of the leading causes of cancer‐related death.^1^ The prognosis of ESCC remains poor despite advances in its diagnosis and treatment, even after esophagectomy and lymph node dissection.^2^ Therefore, a thorough investigation of the cellular and molecular mechanisms that initiate tumorigenesis and promote tumor progression is imperative to provide a better understanding of the medical therapies of ESCC.

Epithelial‐mesenchymal transition (EMT) is a dedifferentiation program in which cells lose their epithelial features and gain migratory behavior, enabling movement away from the epithelial cell community to the surrounding tissue.^3^ EMT is also associated with tumor recurrence and metastasis by enhancing cell motility, as well as with poor patient prognosis.^4^ The characteristic of EMT is that cells acquire mesenchymal cell markers (eg, N‐cadherin and vimentin) and lose epithelial cell markers (eg, E‐cadherin).^5^, ^6^ EMT occurs during the progression of tumors of various origins, including prostate, breast, hepatic, gastric, pancreatic, and colorectal cancer.^7^, ^8^, ^9^, ^10^, ^11^, ^12^

Recently, it has been reported that ESCC possesses EMT characteristics,^13^ and a better comprehension of the role of EMT in tumor invasion and migration will provide additional strategies for the treatment of this disease.

Octamer‐binding transcription factor 4 (OCT4) is a member of the POU‐domain transcription factor family and functions as one of the most important stem cell transcription factors in regulating cancer invasion, migration, and self‐renewal properties.^14^ Previous studies have illustrated that the expression level of OCT4 is increased in ESCC and significantly related to tumor invasion and migration and to poor patient prognosis.^15^ OCT4 may also play a crucial role in EMT and tumor metastasis via enhancing vascular endothelial growth factor C (VEGF‐C) promoter activity to promote VEGF‐C expression and activate VEGF receptor 3 (VEGFR‐3) in ESCC cells.^16^ More studies are needed to demonstrate the function of OCT4 in promoting EMT in ESCC.

Lymphoid enhancer‐binding factor 1 (LEF1), a member of the T‐cell factor (TCF)/LEF1 family of high‐mobility transcription factors, is predominantly contained in the Wnt/β‐catenin signaling pathway, which regulates tumorigenesis and the progression of multiple neoplasms.^17^ The canonical Wnt/β‐catenin pathway is a highly conserved developmental pathway that regulates cancer cell proliferation, differentiation, organ development, and cellular apoptosis.^18^ In addition, LEF1 modulates the interaction with EMT marks, such as E‐cadherin, N‐cadherin, Slug, Twist1, and Snail,^19^ facilitating the process of EMT in hepatocellular carcinoma, MDCK cells, and prostate cancer cells.^20^ Nevertheless, there are limited studies on the role of LEF1 in ESCC.

As mentioned above, several studies have reported the increased expression of OCT4 and LEF1 in many tumor types. This increased expression in cancer suggests that OCT4 and LEF1 might play important roles in tumor proliferation, angiogenesis, invasion, and metastasis. More importantly, previous studies have reported the crosstalk between OCT4 and the components of the Wnt/β‐catenin signaling pathway. For example, as one of the components in the Wnt/β‐catenin signaling pathway, LEF1 mediates the effects of OCT4 in hepatocellular carcinoma cells undergoing EMT.^21^ These findings highlight the potential importance of crosstalk between these two pathways. However, the potential roles of OCT4 and LEF1 have not been reported in ESCC. This study examined the crosslink between OCT4 and LEF1 and analyzed the prognostic relevance of these two genes in patients with ESCC. Additionally, the potential mechanisms of their function in EMT and in tumor invasion and migration were investigated in an ESCC cell line.

## 2 MATERIALS AND METHODS

### 2.1 Patients and tissue specimens

Ninety‐five patient specimens were collected from patients who were diagnosed with primary ESCC and who received radical esophageal surgery without preoperative chemoradiotherapy from 2012 to 2013 at Changhai Hospital (Shanghai, China). All samples were fixed in 4% formaldehyde for 24 hours and embedded in paraffin wax. The patient samples were obtained with informed consent according to an established protocol approved by the Ethics Committee of Changhai Hospital. All patients were observed until May 2017, with a median observance time of 27 months.

### 2.2 Cell culture

Eca109 cells were purchased from the Cancer Cell Repository (Shanghai Cell Bank, Shanghai, China, TCHu 69) and were maintained in high concentration of glucose DMEM supplemented with 10% (v/v) heat‐inactivated fetal bovine serum (Gibco‐BRL) and antibiotics (100 U/mL penicillin and 100 U/mL streptomycin; HyClone Laboratories, Inc., USA) at 37˚C in a humidified atmosphere of 5% CO_2_.

### 2.3 Quantitative Real‐time Reverse‐transcription Polymerase Chain Reaction (qRT‐PCR)

Total RNA was isolated from 1 × 10^6^ cells with TRIzol Reagent (Invitrogen, 15596026), following the manufacturer's protocol. cDNA was synthesized using the PrimeScript RT Reagent Kit (TaKaRa Bio, RR036Q) according to the manufacturer's instructions. Real‐time PCR was performed on a Roche Light Cycler 480 (Roche) using SYBR Premix EX Taq (TaKaRa Bio, 638320). The primer sequences for the OCT4 gene were 5′‐GTACTCCTCGGTCCCTTTCC‐3′ (forward) and 5′‐CAAAAACCCTGGCACAAACT‐3′(reverse); the primer sequences for the LEF1 gene were 5′‐AACATGGTGGAAAACGAAGC‐3′ (forward) and 5′‐GGGTTGGCAGTGATTGTCTT‐3′ (reverse); the primer sequences for E‐cadherin were 5′‐CGAGAGCTACACGTTCACGG‐3′ (forward) and 5′‐GGGTGTCGAGGGAAAAATAGG‐3′ (reverse); the primer sequences for N‐cadherin were 5′‐TTTGATGGAGGTCTCCTAACACC‐3′ (forward) and 5′‐ ACGTTTAACACGTTGGAAATGTG‐3′ (reverse); and the primer sequences for the GAPDH gene were 5′‐ TCAAGAAGGTGGTGAAGCAG‐3′ (forward) and 5′‐ GAGGGGAGATTCAGTGTGGT‐3′ (reverse). GAPDH served as an internal control. The following amplification conditions were used as follows: 1 cycle of 95°C for 30 seconds and 40 cycles of 95°C for 5 seconds and 60°C for 30 seconds. The relative expression level of the target genes was calculated by the 2^−ΔΔCT^ method. All experiments were performed in triplicate.

### 2.4 Western Blot

250 μL 0.1% SDS and 2.5 μL PMSF (phenylmethylsulfonyl fluoride, 1 mmol/L) were applied to collect total protein from 2 × 10^6^ cells. In addition, membrane proteins were extracted using CelLytic^™^ MEM Protein Extraction Kit (Sigma, CE0050), following manufacturer's instructions. To equalize the volume and quantity of protein loaded in each well, we detected protein concentration with a BCA kit (Beyotime, Shanghai, China) and diluted the proteins to the same concentration with PBS. A total of 50 μg of protein was separated by 10% or 7.5% SDS and then transferred to a PVDF membrane (pore size, 0.2 μm) via electroblotting. To block nonspecific antibody binding, the membranes were incubated in 5% nonfat dry milk for 1 hour. Subsequently, the membrane was incubated overnight with GAPDH antibody (Abcam, ab8245, 1:500), human OCT4 antibody (CST, 2750s,1:1000), human LEF1 antibody (Abcam, ab137872, 1:1000), E‐cadherin antibody (Abcam, ab40772, 1:10 000), N‐cadherin antibody (Abcam, ab18203, 1:1000), and Na^+^/K^+^ ATPase (Santa Cruz, SC‐21712, 1:500). Dilute primary antibodies with primary antibody dilution buffer (Beyotime Biotechnology, Dalian, China, P0023A). Goat anti‐mouse or goat anti‐rabbit secondary antibodies (CST, 7076,7074, 1:4000) were incubated with the membranes for 1 hour at room temperature. The target genes were then detected by enhanced chemiluminescence reagent. Finally, we compared the protein expression level of OCT4, LEF1, E‐cadherin, and N‐cadherin with that of GAPDH in each group using the Image‐Pro Plus 6.0 system (Bio‐Rad, 1708265). The results were measured by ImageJ software (version: 1.34). All experiments were performed in triplicate.

### 2.5 Immunohistochemistry (IHC)

A total of 95 esophageal squamous cell carcinoma samples were fixed in 4% methanol, embedded, and sliced to a thickness of 5 μm. After deparaffinization and rehydration procedures, epitope retrieval was executed in heated citrate buffer (pH 6.0) for 30 minutes as recommended. Then, we incubated the slides with the UltraSensitive Streptavidin‐Peroxidase Kit (Fuzhou Maixin Biotechnology, Kit‐9710, Fuzhou, China) and the following primary antibodies: OCT4 (1:50, Santa Cruz, sc‐5279) and LEF1 (5 μg/mL, Abcam, ab137872). Nonspecific binding sites were blocked by incubation with 5% rabbit serum for 10 minutes. DAB (Fuzhou Maixin Biotechnology, DAB‐0031) was applied to manifest specific markers, and counterstaining was performed with hematoxylin. The immunostaining staining levels were evaluated as 0 (negative), 1 +  (weak positive), 2 +  (moderate positive), and 3 +  (strong positive). High expression level was defined as a score ≥2. All experiments were performed in triplicate.

### 2.6 Construction and transfection of shRNA vectors, lentivirus, and overexpression plasmids

The shRNA sequence for human OCT4 (shOCT4: 5′‐ GGTGCTCGATAAATCTCTTGA‐3′) was encoded in the lentiviral expression plasmid pHBLV‐U6‐ZsGreen‐Puro vector (Hanheng Bio‐technology Co., Shanghai, China), in which the encoded sequences were controlled by the U6 promoter. The lentivirus was produced by directly transfecting the loading plasmids and the transfer lentiviral plasmids into Eca109 cells. After 48 hours, >80% efficiency of infection was observed according to green fluorescent protein. The overexpression plasmid pcDNA3.1‐LEF1 was purchased from the Heyuan Bio‐technology Company (Shanghai, China). The LEF1 overexpression plasmid was transfected with Lipofectamine 2000 (Invitrogen; Thermo Fisher Scientific Inc., USA), and target gene expression was observed after 48 hours of culture.

### 2.7 Invasion assay

Cell invasion was examined by transwell invasion assay with a chamber pore size of 8 μm (Corning). The transwell filter inserts were coated with Matrigel (Corning). A total of 5 × 10^4^ cells were seeded in serum‐free medium in the upper chamber. After 24 hours of incubation in 37°C, cells in the upper chamber were carefully removed with a cotton swab, and cells remaining on the membrane were stained with 0.1% crystal violet solution and counted. The results were measured by ImageJ software (version: 1.34). All experiments were performed in triplicate.

### 2.8 Migration assay

Transwell migration chambers were used to detect cancer cell migration ability. A total of 5 × 10^4^ transfected Eca109 cells were plated in the upper chamber (pore size, 8 μm; Corning) without Matrigel. The cells were then incubated for 24 hours. Cells in the upper chamber were carefully removed with a cotton swab, and the remaining cells were stained with 0.1% crystal violet solution and counted. The results were measured by ImageJ software (version: 1.34). All experiments were performed in triplicate.

### 2.9 Wound‐healing assay

The cells were plated on 6‐well plates and grown to 90% confluence. Adherent cells in the center of the well were scraped with pipette tips and washed with PBS. Cell movement into the wound area was captured at 0 and 24 hours using a light microscope. The migration areas between the front edge of the migrating cells and the edge of the wound were compared using ImageJ software. The results were measured by ImageJ software (version: 1.34). All experiments were performed in triplicate.

### 2.10 Statistical analysis

SPSS 22 software (SPSS, Chicago, IL, USA) was used to statistically analyze the data. The quantitative data were first evaluated whether they followed the normal distribution by the Shapiro‐Wilk test. Two‐tailed Student's *t* test was applied to datasets with normal distribution. When appropriate in case of multiple comparisons, two‐tailed ANOVA with LSD post‐test was applied. Data were expressed as mean±SD. The association between markers and clinicopathological features and the relationship between relative gene expression and clinical pathological data were analyzed by chi‐square test, Fisher's exact test, or *t* test. Pearson's rank correlation was used to analyze the correlation of the expression of both genes. Survival curves were analyzed using the Kaplan‐Meier method. The experimental data were obtained in three independent experiments. *P* < .05 indicated statistical significance.

## 3 RESULTS

### 3.1 Elevated expression of both OCT4 and LEF1 indicates poor prognosis and is associated with tumor biological characteristics in patients with ESCC

**Figure 1:**
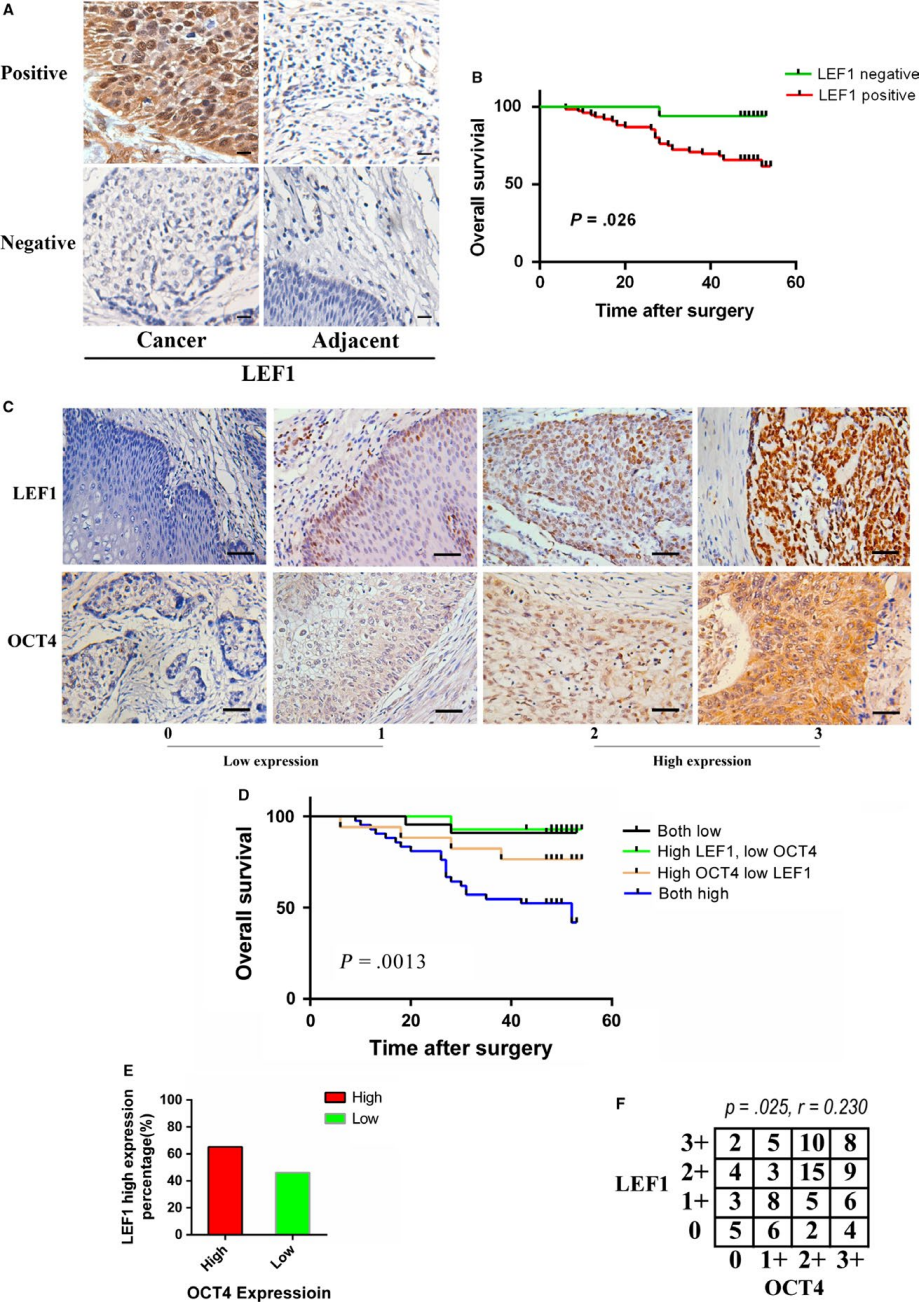
OCT4 and LEF1 expressions in ESCC tissue were determined by immunochemistry. A, LEF1 expression in ESCC cancer tissue compared with adjacent normal tissue (scale bar = 20 μm). B, Overall survival curve of patients with negative LEF1 expression (green line) and patients with positive LEF1 expression (red line), *P *=* *.026. C, Immunochemistry analysis of OCT4 and LEF1 expression. Representative staining intensity of OCT4 and LEF1 represents different expression levels (scale bar = 100 μm). D, The overall survival of 95 patients with ESCC was compared, *P *=* *.0013. E, The percentage of patients with high LEF1 staining was higher in the OCT4‐high group. F, Correlation of expression levels of OCT4 and LEF1 in ESCC measured by Pearson's rank correlation analysis. Pearson's *r* = .230, *P *=* *.025

Previous studies have shown that the expression of OCT4 is increased to a variable extent in tumor specimens from patients with ESCC.^22^ However, no studies have reported on LEF1 expression and its relationship with clinicopathological features in ESCC. In this study, the levels of LEF1 in ESCC and adjacent normal tissue were examined by immunohistochemical analysis. As shown in Figure 1A and Table 1, the expression level of LEF1 was increased in 82.1% (78/95) of patients with ESCC, but weak or no expression was detected in adjacent normal tissue. Clinicopathological analysis results indicated that positive staining for LEF1 was obviously associated with histological grade, TNM stage, and overall survival rate (Table 2, Figure 1B). Previous studies have shown that the overexpression of OCT4 is associated with the poor prognosis of patients with ESCC and with clinicopathological characteristics (gender, age, cell differentiation, invasion path, and lymph node metastasis).^23^ In this study, we investigated the relationship between OCT4 and LEF1 expression in the same 95 patient specimens. Based on the expression levels of these proteins, all 95 patients were classified into 4 groups: group I (n = 42), high OCT4 and LEF1 intensity; group II (n = 17), high OCT4 but low LEF1 intensity; group III (n = 14), high LEF1 but low OCT4 intensity; and group IV (n = 22), low OCT4 and LEF1 intensity (Figure 1C). In the OCT4‐high ESCC groups, the percentage of patients with high LEF1 was 71.2% higher than that in the OCT4‐low groups, indicating an association between OCT4 and LEF1 expression (Figure 1E). Moreover, the OCT4^high^/LEF1^high^ group exhibited aggressive clinicopathological features, including advanced histological grade and TNM stage and the presence of lymph node metastasis (Table 3). Additionally, the correlation of OCT4 and LEF1 expression in the same tissue by utilizing Pearson's rank correlation revealed that the aberrant expression of OCT4 was positively associated with the overexpression of LEF1 in ESCC (Figure 1F). More importantly, Kaplan‐Meier analysis results indicated that concomitant elevated expression of OCT4 and LEF1 could predict prognosis for patients with ECSS. (Figures 1D and S1). Therefore, the combination of high OCT4 and LEF1 expression can be a prognostic predictor for ESCC.

**Table 1:** Expression of LEF1 in ESCC tumor tissues and corresponding adjacent normal tissues

	Cases	LEF1 expression, n (%)		*P* value
		Negative	Positive	
Tumor tissues	95	17(17.9)	78(82.1)	**<.01**
Normal tissues	95	60(63.2)	35(36.8)	

Bold value highlighted the significant statistical results.

**Table 2:** Correlation between LEF1 expression and clinicopathological characteristics in 95 patients

	LEF1		
	Negative (17)	Positive (78)	*P* value^a^
Sex			
Male	14	64	1.01
Female	3	14	
Age (y)^b^			
>65	5	18	.755
≤65	12	60	
T grade			
1	10	19	**.02**
2	5	23	
3	2	34	
4	0	2	
Lymph node metastasis			
N0	12	43	.213
N1	1	21	
N2	1	13	
N3	0	4	
TNM stage			
I	10	13	**<.01**
II	5	23	
III	2	42	
Death			
Yes	1	14	**<.01**
No	16	64	

a Statistical significance was determined by chi‐square test or Fisher's exact test.

b Data are presented as the mean ± SD.

Bold value indicated the significant statistical results to distinguish the insignificant results.

**Table 3:** Clinicopathological characteristics by OCT4 and LEF1 expression

	OCT4/LEF1 expression				*P* value^a^
	Both high (42)	High‐OCT4, Low‐LEF1 (17)	High‐LEF1, Low‐OCT4 (14)	Both Low (22)	
Sex					
Male	35	11	13	19	.175
Female	7	6	1	3	
Age (y)^b^					
>65	9	5	4	5	.931
≤65	33	12	10	17	
T grade					
1	6	5	5	11	.17
2	9	6	6	5	
3	26	5	3	6	
4	1	1	0	0	
Lymph node metastasis					
N0	17	9	9	20	**.23**
N1	12	5	4	1	
N2	10	2	1	1	
N3	3	1	0	0	
TNM stage					
I	5	4	2	12	**.16**
II	12	6	3	7	
III	25	7	9	3	
Death					
Yes	23	4	1	2	**<.01**
No	19	13	13	20	

a Statistical significance was determined by chi‐square test or Fisher's exact test for categorical/binary measures and by ANOVA for continuous measures.

b Data are presented as the mean ± SD.

Bold value indicated the significant statistical results to distinguish the insignificant results.

### 3.2 OCT4 is positively regulated with LEF1 expression in Eca109 cells in vitro

**Figure 2:**
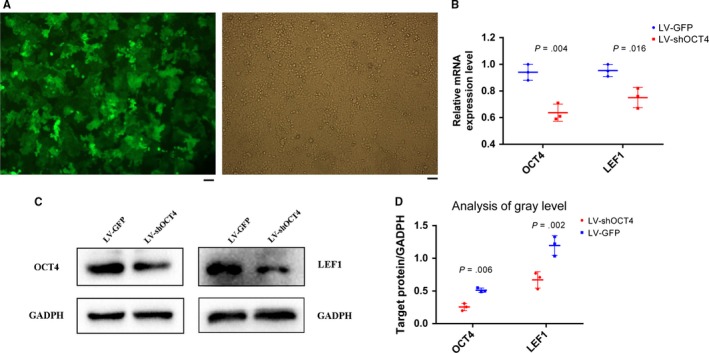
Verification that OCT4 positively regulated LEF1 expression in Eca109 cells. A, The transfection efficiency of the lentiviral system in Eca109 cells, evaluated by fluorescence microscopy, reached ≥90% (scale bar = 50 μm). B, Relative mRNA expression level was significantly decreased in the LV‐shOCT4 group by two‐tailed *t* test (n = 3 for each cell group). C, Protein levels of OCT4 and LEF1 in the LV‐shOCT4 group were significantly downregulated by two‐tailed *t* test. D, Analysis of gray level between each group was applied by ImageJ software. All experiments were performed in triplicate

As the high OCT4 and LEF1 expression levels in 95 patients with ECSS showed a correlation with tumor pathogenesis and poor clinicopathological characteristics, we further explored the relationship between OCT4 and LEF1 in vitro. To this end, we applied lentiviral constructs expressing small hairpin RNA targeting OCT4 (LV‐shOCT4) to infect Eca109 cells, and the infection efficiency reached ≥80% (Figure 2A). The cells were infected with GFP‐shRNA (LV‐GFP) as a negative control. The expression level of OCT4 and LEF1 was examined 48 hours later. The OCT4 relative mRNA level in the LV‐shOCT4 group was 0.636 compared with that in the LV‐GFP group (Figure 2B). More importantly, the LEF1 relative mRNA level in the LV‐shOCT4 group was simultaneously decreased, indicating the positive regulation of LEF1 by OCT4. Similarly, Western blot results also showed the decreased expression of OCT4 and LEF1 proteins in the LV‐shOCT4 group (Figures 2C,D, and S2). Taken together, these findings indicate that LEF1 is positively regulated by OCT4 at the transcription level in Eca109 cells.

### 3.3 LEF1 mediates the effects of OCT4 in the EMT, invasion, and migration of ESCC

**Figure 3:**
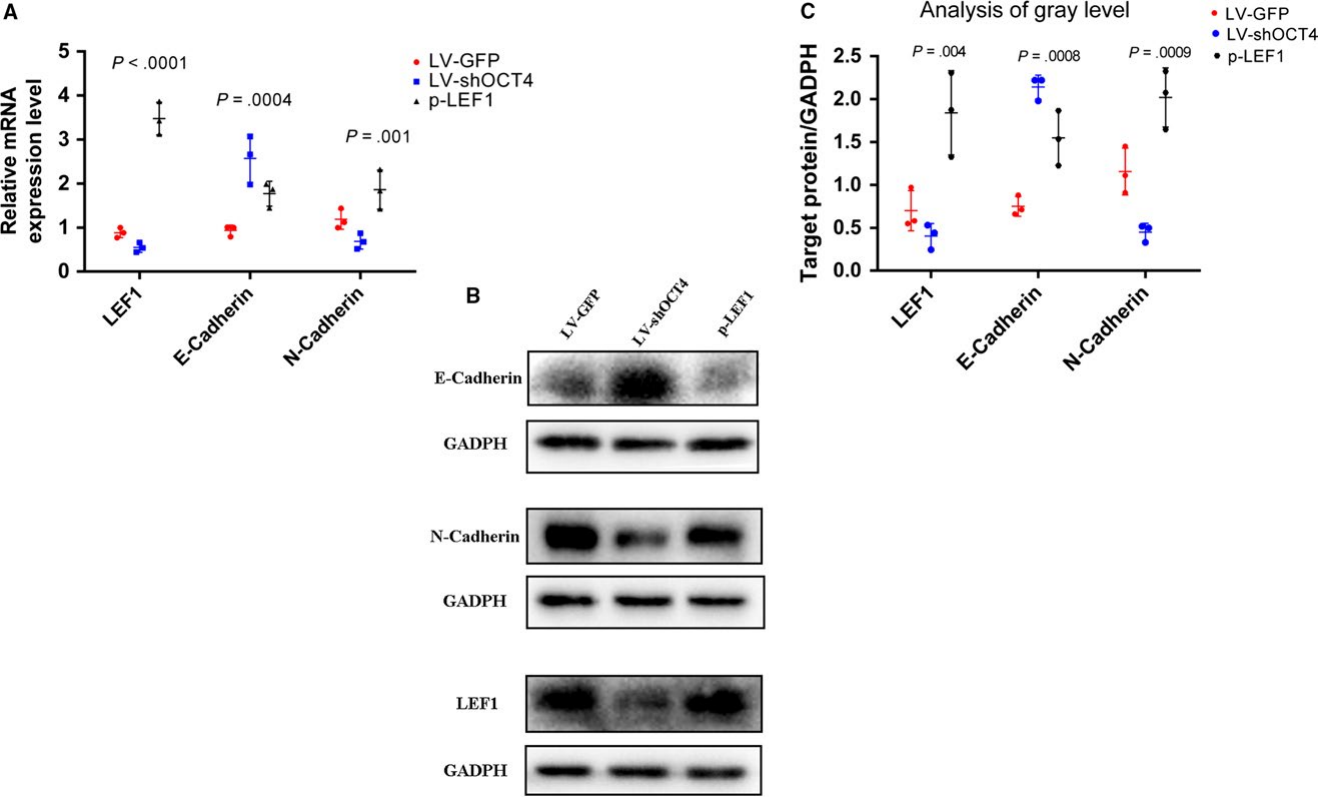
LEF1 was a facilitator of OCT4 in regulating the EMT, invasion, and migration of Eca109 cells. A, Relative mRNA expression level: successful overexpression of LEF1 in the LV‐shOCT4 group; overexpression of N‐cadherin and low expression of E‐cadherin in the p‐LEF1 group, two‐tailed ANOVA was used and LSD test was significant between shOCT4 and p‐LEF1 groups. B and C, The protein level in the p‐LEF1 group enhanced expression of N‐cadherin and decreased expression of E‐cadherin. Analysis of gray level was performed by ImageJ software, two‐tailed ANOVA was used, LSD test was significant between shOCT4 and p‐LEF1 groups, and LSD test was significant between shOCT4 and p‐LEF1 groups

**Figure 4:**
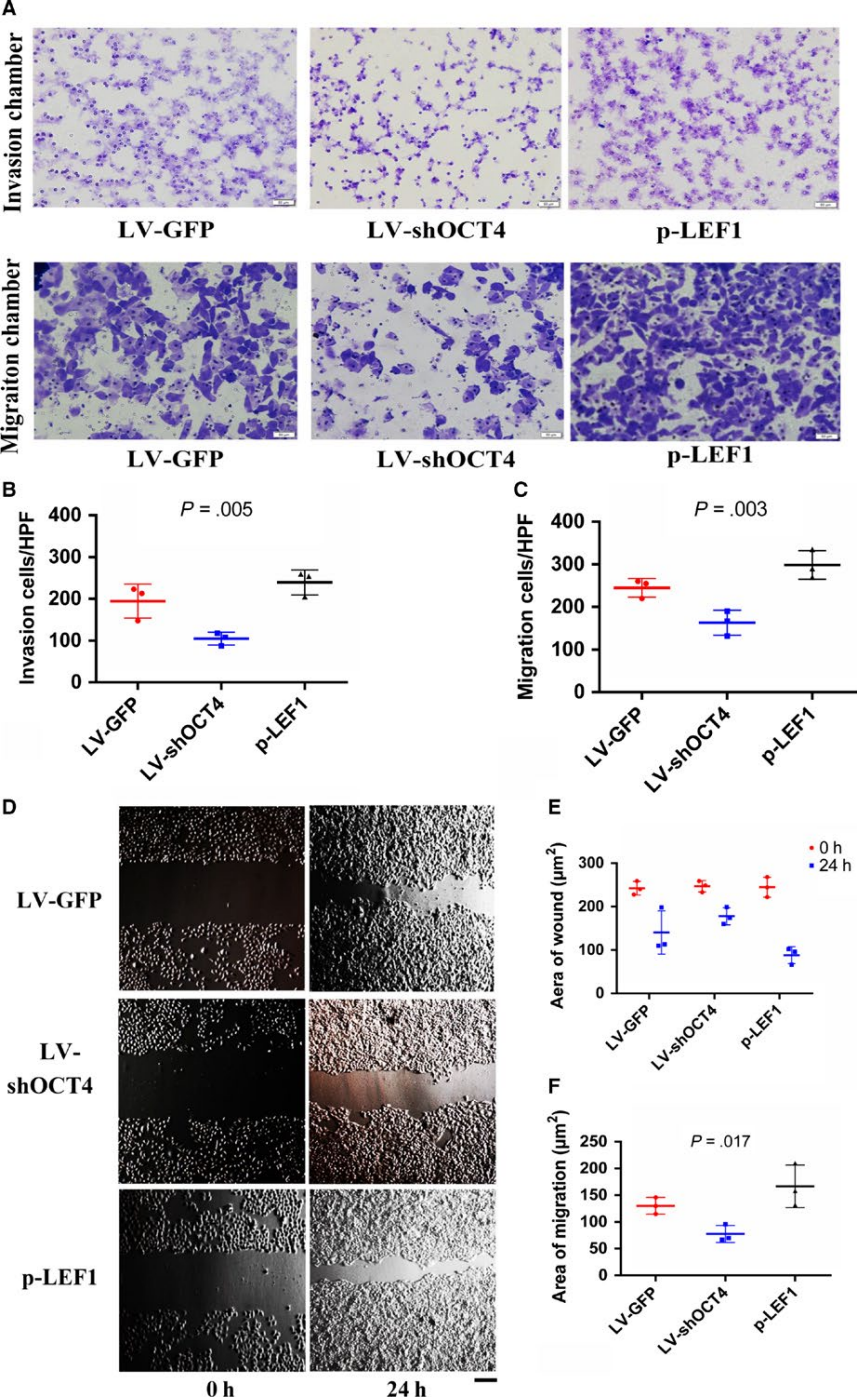
LEF1 is a facilitator of OCT4 in regulating the invasion and migration of Eca109 cells (scale bar = 50 μm). A and B, In the transwell assay, the p‐LEF1 group showed a higher invasion ability. Quantified data are presented as the number of invaded cells per HPF (n = 3 for each cell group), two‐tailed ANOVA was used, and LSD test was significant between shOCT4 and p‐LEF1 groups. A and C, In the transwell assay, the p‐LEF1 group showed a higher migration ability. Quantified data are presented as the number of migrated cells per HPF (n = 3 for each cell group), two‐tailed ANOVA was used, and LSD test was significant between shOCT4 and p‐LEF1 groups. D, Wound‐healing assays showed greater migration ability in the p‐LEF1 group. E and F, Quantified data are presented as the wound area and the area covered by migrating cells per HPF (n = 3 for each cell group), two‐tailed ANOVA was used, and LSD test was significant between shOCT4 and p‐LEF1 groups. All experiments were performed in triplicate

Considering that LEF1 is reportedly involved in cell EMT, invasion, and migration, it was necessary to investigate whether the effects of OCT4 on tumor EMT, invasion, and migration are mediated by LEF1 in the Eca109 cell line. First, we constructed a LEF1 overexpression plasmid to apply a rescue strategy. To this end, we transfected the LEF1 overexpression plasmid into LV‐shOCT4 Eca109 cells (p‐LEF1). Western blotting and qRT‐PCR results showed that OCT4 and LEF1 expression levels were decreased in LV‐shOCT4 Eca109 cells (Figures 2B,C and S2), while LEF1 expression was significantly increased in p‐LEF1 Eca109 cells compared with shOCT4 cells (Figures 3A‐C and S3). Furthermore, we evaluated the role of LEF1 in facilitating the effects of OCT4 on tumor cell EMT, invasion, and migration. The results of Western blotting and qRT‐PCR revealed decreased levels of E‐cadherin and increased levels of N‐cadherin in the p‐LEF1 group, indicating enhanced EMT capacity (Figures 3A,B, and S1D, S3,S4). In addition, the results of transwell invasion and migration chamber assays showed that the group of LEF1 overexpression in LV‐shOCT4 Eca109 cells presented an increase in not only invasion but also migration (Figure 4A‐C). Moreover, wound‐healing assay also showed increased migration ability in the p‐LEF1 group (Figure 4D,E). However, knocking down OCT4 did not enhance the EMT, invasion, and migration abilities in the LV‐shOCT4 group. These results revealed that the regulatory effects of OCT4 on EMT, invasion, and migration in ECA109 cells were, at least partially, mediated by LEF1.

## 4 DISCUSSION

OCT4, as an important transcription factor, participates in regulating self‐renewal and pluripotency in undifferentiated embryonic stem cells.^24^ This protein has gradually become a hopeful biomarker for the diagnosis of many tumors.^25^ As previously mentioned, OCT4 participates in tumorigenicity and tumor progression in many somatic cell cancers, including esophageal, bladder, prostate, gastric, and non‐small‐cell lung cancers. OCT4 plays an important role in a series of signaling pathways, such as the Wnt/β‐catenin, TGF‐β, and JAK/STAT3 signaling pathways,^26^ to enhance or inhibit downstream target genes. In particular, the Wnt/β‐catenin pathway is a highly conserved developmental signaling pathway implicated in controlling cancer cell proliferation, differentiation, and EMT.

LEF1 is a transcription factor that primarily participates in the Wnt/β‐catenin signaling pathway. LEF1 is also a facilitator of EMT, a feature of cancer cell migration and invasion, as well as cancer cell proliferation and viability.^17^ Undoubtedly, the crucial position of LEF1 in cancer progression makes this protein an ideal biomarker for predicting patient prognosis, which shows the value of its clinical application. Moreover, the indispensable role of LEF1 in propagating cancerous growth and metastasis also suggests that LEF1 is an ideal target for therapeutic treatment of cancer progression. For example, knockdown of LEF1 reduced colon cancer cell (SW480 and SW620) invasion by decreasing MMP‐2 and MMP‐9 expression.^27^ However, the current existing LEF1‐targeted therapy did not completely diminish cancer cell growth, but it helps promise in combination with multiple targeted agents. Thus, there is an urgent need to adequately understand the regulatory mechanism between OCT4 and LEF1.

A previous study showed that overexpression of OCT4 in ESCC has been consistently associated with cancer proliferation, metastasis, and drug resistance.^22^ In the present study, based on the analysis of 95 ESCC patient samples with complete follow‐up data, the LEF1 expression level in ESCC was higher than that in adjacent tissue, and the high expression of LEF1 in patients was strongly associated with poor prognosis. Moreover, we detected both OCT4 and LEF1 expressions in ESCC tumor specimens and found that the combination of OCT4 and LEF1 was closely related to the surgical outcomes of patients with ESCC. Patients with OCT4^high^/LEF1^high^ tumors had a poorer prognosis and more aberrant clinicopathological features than the high/low and low/low expression groups, and Spearman's rank correlation results showed that the aberrant expression of LEF1 was positively associated with the overexpression of OCT4 in ESCC. Therefore, the present study showed that the union of OCT4 and LEF1 was strongly related to the poor prognosis of patients with ESCC, but the regulatory mechanisms between OCT4 and LEF1 in ESCC remain unknown.

OCT4 can activate multiple signaling pathways, such as the Wnt/β‐catenin signaling pathway, to control LEF1 expression. A previous study revealed that in hepatocellular carcinoma, OCT4 activates the LEF1/β‐catenin‐dependent Wnt signaling pathway to induce cancer cell EMT.^21^ Moreover, Tcf/Lef‐Oct4 composite element can bond to the promoter of Mesp1. The cooperation between OCT4 and Wnt signaling thus plays an important role in Mesp1 expression and cardiac program. This composite may drive pluripotency via SOX2‐OCT4 and switch on lineage‐related genes through Oct4's recruitment of Tcf/Lef factors.^23^ As a high level of OCT4 expression was detected in the Eca109 cell line,^28^ we inhibited the expression of OCT4 via the lentiviral infection system with plasmid vectors encoding OCT4‐shRNA. The results indicated that OCT4 positively regulated LEF1 expression. Furthermore, we applied a rescue strategy to detect the relationship between OCT4 and LEF1 in ESCC. A LEF1 overexpression plasmid vector was utilized to transfect LV‐shOCT4‐expressing Eca109 cells, resulting in LEF1 overexpression in OCT4^low^ cells. Additionally, we implemented qRT‐PCR, Western blotting, flow cytometry, and wound‐healing assays to detect the ability of LEF1 to mediate the effects of OCT4 in facilitating EMT, invasion, and migration. Expectedly, the p‐LEF1 group exhibited more marked EMT, invasion, and migration than the LV‐shOCT4 and negative control groups. Thus, these results demonstrated that OCT4 positively regulated LEF1 expression, and LEF1 mediated the effects of OCT4 in the EMT, invasion, and migration in ESCC.

The present study showed that the overexpression of both OCT4 and LEF1 is an important feature in ESCC progression, and OCT4 is closely correlated with LEF1 expression in the regulation of cancer cell EMT, invasion, and migration. However, the molecular mechanisms between OCT4 and LEF1 are complicated. Mesp1, a cardiac mesoderm gene, may be regulated by LEF1‐OCT4 composite. The activation of Mesp1 gene in embryonic stem cells (ESCs) may lead to commitment to the cardiac program.^23^ However, there are no relative studies of the function of Mesp1 in ESCC, which is a hopeful direction. Additional studies are needed to verify the genetic characteristics of OCT4 and LEF1.

## CONFLICT OF INTEREST

The authors declare that they have no financial and personal relationships with other individuals or organizations that can inappropriately influence this study, and there is no professional or other personal interest of any nature or type in any product, service, and/or company that could be construed as influencing the position presented within, or in the review of, the entitled manuscript.
