# Supplementary material for: Prognostic value of association of OCT4 with LEF1 expression in esophageal squamous cell carcinoma and their impact on epithelial‐mesenchymal transition, invasion, and migration
Source: Cancer Med. 2018 Jul 4;7(8):3977–87. doi: 10.1002/cam4.1641 (PMC6089166; doi:10.1002/cam4.1641)

Supplement Fig.1

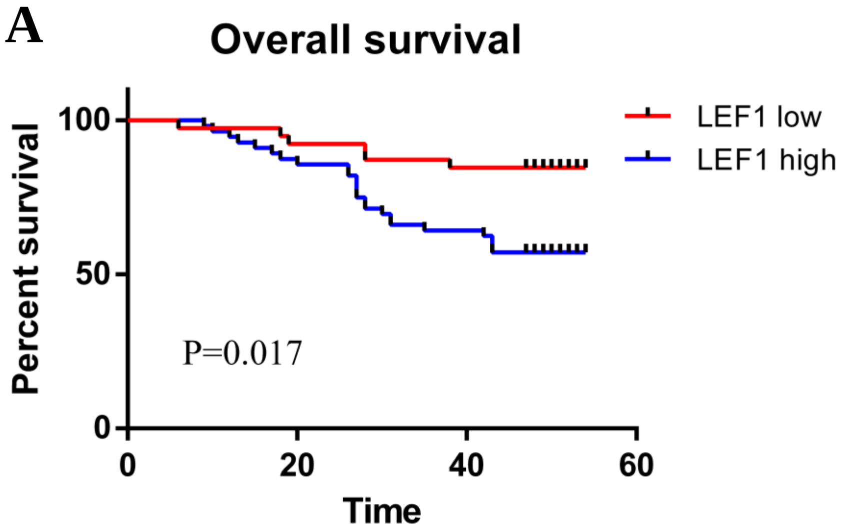

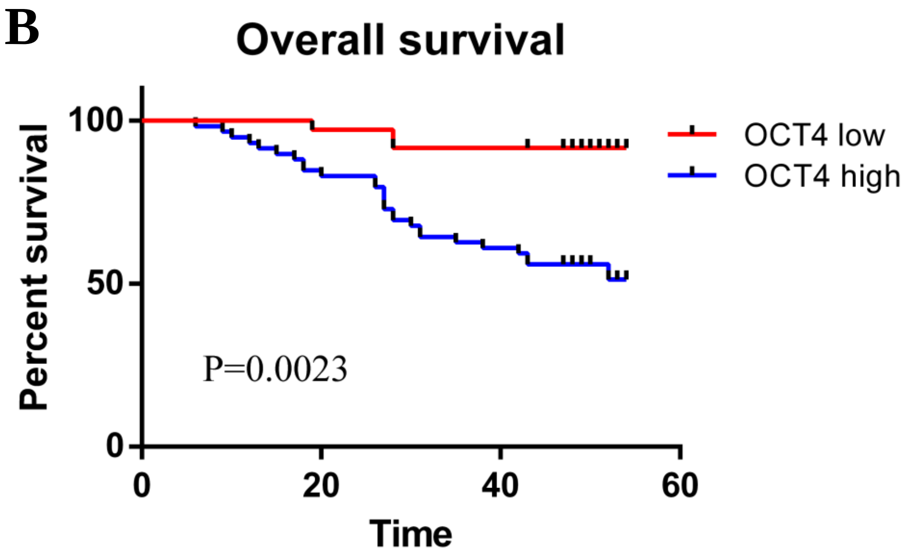

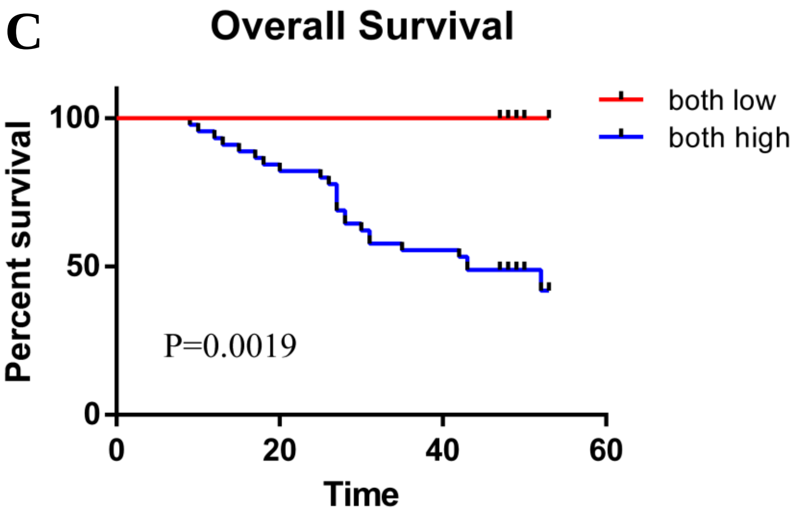

Supplement Fig.1

D

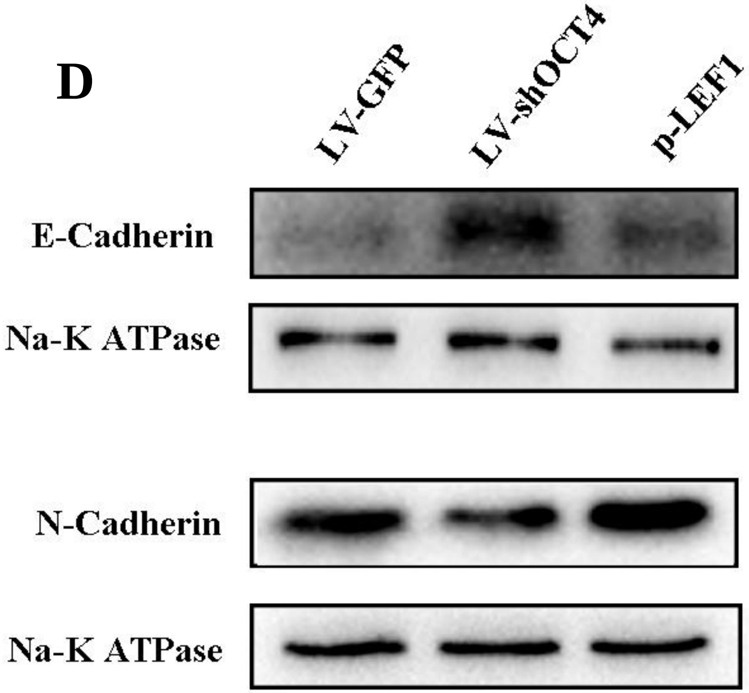

Supplement Fig.2

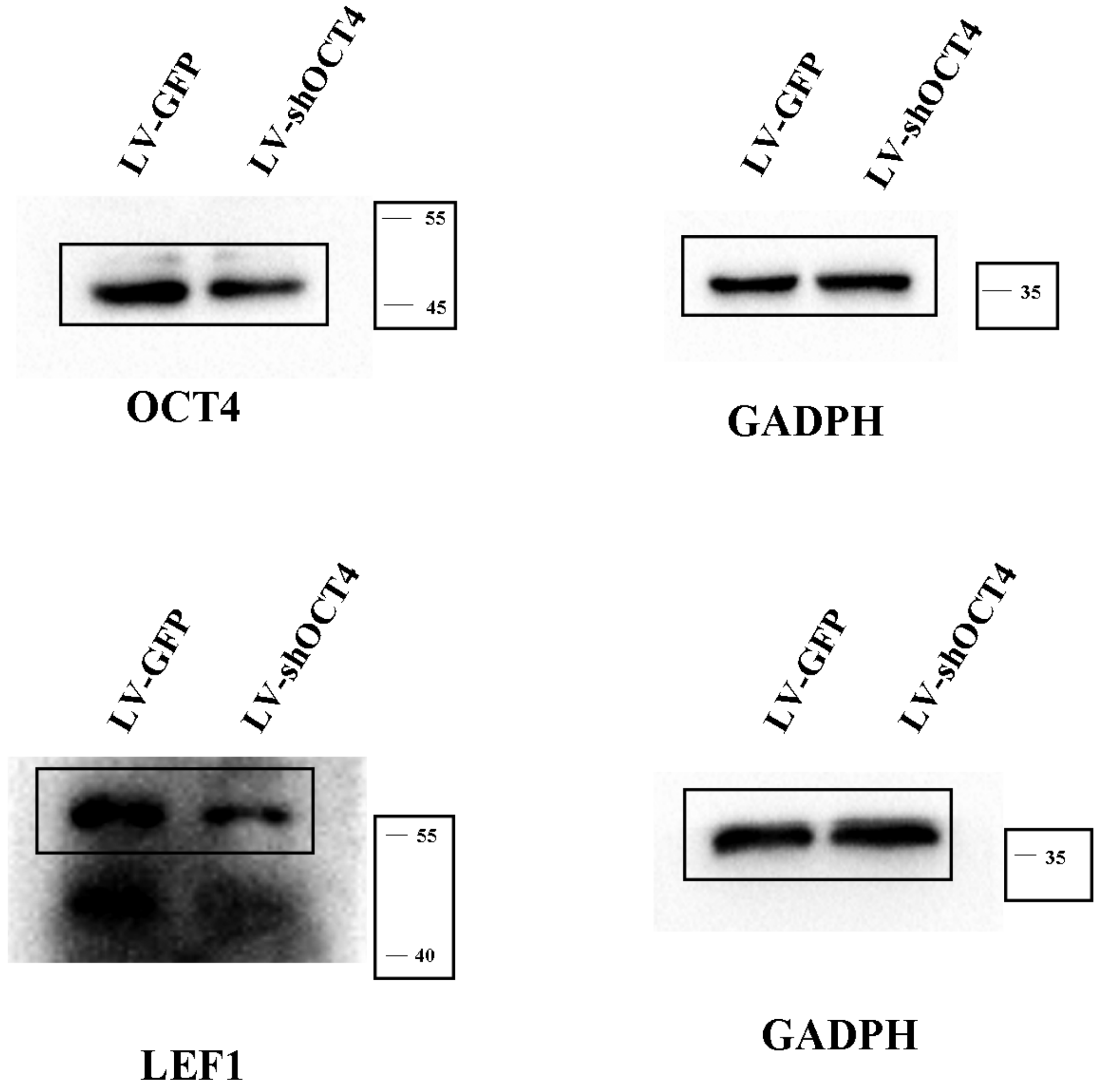

Supplement Fig.3

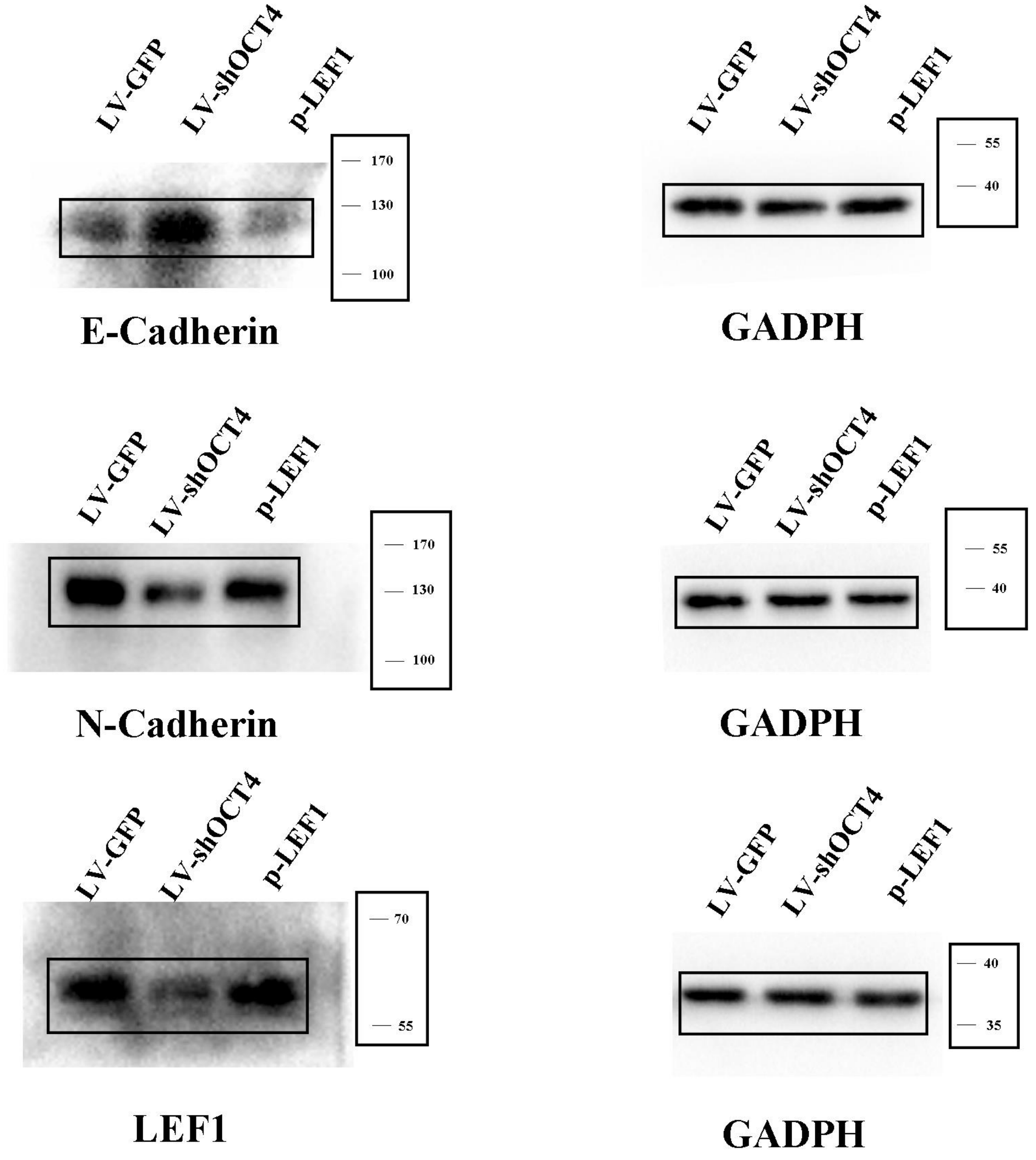

Supplement Fig.4

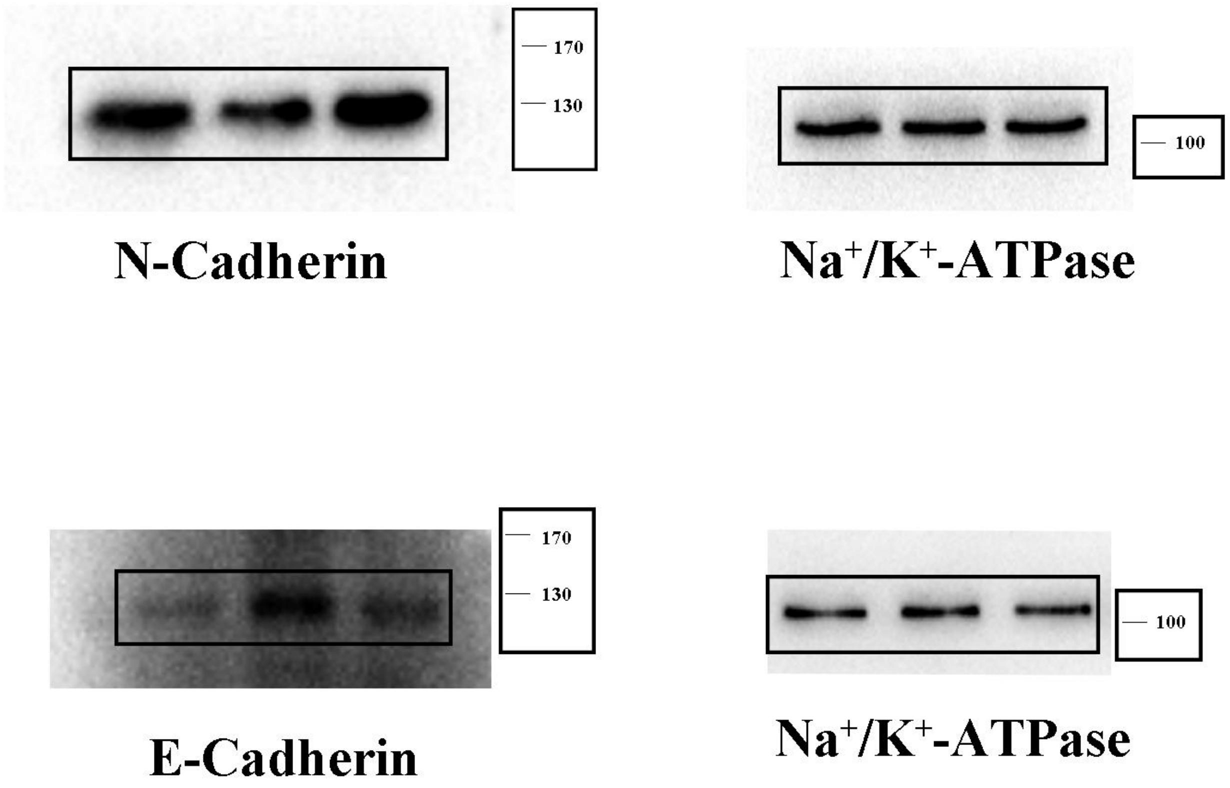

Supplement: Supplementary file 1 [file CAM4-7-3977-s001.pdf]
