# Supplementary material for: Prognostic value of association of OCT4 with LEF1 expression in esophageal squamous cell carcinoma and their impact on epithelial‐mesenchymal transition, invasion, and migration
Source: Cancer Med. 2018 Jul 4;7(8):3977–87. doi: 10.1002/cam4.1641 (PMC6089166; doi:10.1002/cam4.1641)
Supplement: Supplementary file 2 [file CAM4-7-3977-s002.docx]

**Supplement References**

Li, Y, Yu, W, Cooney, A. J, Schwartz, R. J, Liu, Y (2014). Brief report: oct4 and canonical wnt signaling regulate the cardiac lineage factor mesp1 through a tcf/lef-oct4 composite element. Stem Cells, 31(6), 1213-1217. DOI: 10.1002/stem.1362

**Supplement Figure Legend**

**Supplement Fig.1**. (A) Overall survival curve of patients with low LEF1 expression (red line) and patients with high LEF1 expression (blue line), *P*=0.017. (B) Overall survival curve of patients with low OCT4 expression (red line) and patients with high OCT4 expression (blue line), *P*=0.0023. (C) Overall survival curve of patients with both low expression (red line) and patients with both high expression (blue line), *P*=0.0019. (D). The membrane protein level in the p-LEF1 group, enhanced expression of membrane N-cadherin and decreased expression of membrane E-cadherin.

**Supplement Fig.2**. Uncropped whole-membrane immunoblots with protein marker size are presented in the same order as in the corresponding figures. Protein levels of OCT4 and LEF1 in the LV-shOCT4 group were significantly downregulated.

**Supplement Fig.3**. Uncropped whole-membrane immunoblots with protein marker size are presented in the same order as in the corresponding figures.

**Supplement Fig.4**. Membrane E-Cadherin and N-cadherin uncropped whole-membrane immunoblots with protein marker size are presented in the same order as in the corresponding figures.
